# Pharmacologic targeting of midasin (MDN1) reveals a potential therapeutic vulnerability in ESR1-mutant breast cancer

**DOI:** 10.3389/fphar.2026.1852788

**Published:** 2026-07-17

**Authors:** Mounika Pamukuntla, Afia Ohemeng, Bipika Banjara, Manasa Kotina, Jillian L. Pope, Simak Ali, Selina Darling-Reed, Matthew E. Burow, Madhavi Gangapuram, Suresh Eyunni, Jaylen Thomas, Aaron Hilliard, A. Michael Davidson, Elizabeth Henderson, Syreeta L. Tilghman

**Affiliations:** 1 Division of Basic Pharmaceutical Sciences, College of Pharmacy and Pharmaceutical Sciences, Institute of Public Health, Florida A&M University, Tallahassee, FL, United States; 2 Department of Biological Sciences, College of Science and Technology, Tallahassee, FL, United States; 3 Department of Surgery and Cancer, Imperial College London Hammersmith Hospital Campus, London, United Kingdom; 4 Section of Hematology and Medical Oncology, Tulane Department of Medicine, Tulane University Health Science Center, New Orleans, LA, United States; 5 School of Pharmacy, Hampton University, Hampton, VA, United States

**Keywords:** breast cancer, endocrine resistance, estrogen receptor, estrogen receptor mutations, midasin, ribozinoindoles

## Abstract

**Introduction:**

Although most cases of estrogen receptor-positive (ER+) breast cancer initially respond to endocrine therapy, many patients ultimately develop resistance. A major contributor to endocrine resistance in metastatic disease is the acquisition of constitutively active somatic mutations in the estrogen receptor ligand-binding domain (LBD). We previously identified midasin (MDN1), a ribosome biogenesis protein, as significantly overexpressed in letrozole-resistant MCF-7 cells. Because these cells are ER^low/−^ and represent only a subset of endocrine-resistant tumors, we hypothesized that ESR1 mutations cooperate with MDN1 dysregulation to confer a survival advantage.

**Methods:**

To address this, the cBioPortal database was queried to assess correlations between breast cancer subtypes and MDN1 expression levels. MCF-7 cell lines harboring ER point mutations were evaluated by RNA sequencing and immunoblot analysis to measure ER and MDN1 expression. To identify pharmacologic inhibitors of midasin, computational docking analyses were performed using a panel of ribozinoindole (Rbin) analogs, followed by biological evaluation studies.

**Results:**

Initial analyses demonstrated that MDN1 expression is elevated in human breast cancer tumors, including luminal, HER2+, and triple-negative breast cancer. RNA sequencing of parental MCF-7 cells and ESR1-mutant derivatives, MCF-7 Y537S and MCF-7 D538G, revealed comparable MDN1 transcript levels across all cell lines. In contrast, immunoblot analysis showed mutation-dependent differences in MDN1 protein expression, with the highest levels observed in MCF-7 D538G cells, followed by MCF-7 Y537S cells and then parental MCF-7 cells. Computational docking analyses of Rbin analogs led to the selection of Rbin-1 and Rbin-2 for biological evaluation. Viability assays revealed minimal activity for Rbin-1, whereas Rbin-2 reduced proliferation by 30%–55% across all three cell lines, with the most pronounced effects observed in MCF-7 D538G cells at 24 and 48 h. Consistent with these findings, Rbin-2 treatment decreased MDN1 protein expression by approximately 50% in all cell lines, while ER levels remained largely unchanged.

**Discussion:**

Collectively, these results establish the feasibility of pharmacologically targeting midasin in mammalian cell lines and support a functional link between MDN1 expression and ESR1 mutation-driven endocrine resistance. This work provides a foundation for future mechanistic studies of midasin as a potential therapeutic vulnerability in ER-mutant breast cancer.

## 1 Introduction

Although mortality rates for estrogen receptor positive (ER+) breast cancer in the United States have declined substantially with the advent of effective endocrine therapies (e.g., aromatase inhibitors (AIs), selective estrogen receptor modulators, and CDK4/6 inhibitors), the emergence of therapeutic resistance remains a major clinical challenge. To explore additional mechanisms underlying endocrine resistance, we previously characterized the global proteomic profile of cancer stem cell enriched, letrozole-resistant breast cancer cells and identified increased expression of midasin (MDN1), a protein involved in ribosomal biogenesis (Gallegos et al., 2021). Although these findings provided important insight into pathways associated with AI resistance, they were preliminary in nature and the precise role of midasin in endocrine resistance is unknown. Further, endocrine resistance is highly heterogeneous, and the model system used represented ER^low/–^ letrozole-resistant cells, which comprise only a subset of resistant breast tumors. Therefore, understanding the biological function of midasin and how its dysregulation may influence cellular growth and proteostasis is critical for evaluating its potential contribution to *de novo* or acquired endocrine-resistant breast cancer phenotypes.

Midasin is a conserved AAA + ATPase that functions as a mechanochemical enzyme in late-stage ribosome biogenesis, with a specific role in maturation of the 60S ribosomal subunit. It operates predominantly in the nucleus and nucleolus, where it facilitates structural remodeling of pre-60S particles (Chen et al., 2018; Erzberger and Berger, 2006; Garbarino and Gibbons, 2002). Through ATP hydrolysis, midasin promotes the dissociation of defined assembly factors from immature ribosomal subunits, a prerequisite for acquisition of export competence and functional maturation (Hanson and Whiteheart, 2005). Perturbation of midasin expression or activity disrupts 60S subunit biogenesis and compromises ribosome output. This defect leads to reduced translational capacity and induction of nucleolar stress responses, which can engage cell-cycle checkpoint pathways and growth arrest programs. Conversely, increased midasin activity may enhance ribosome biogenesis and protein synthesis, thereby altering proteostasis and conferring a proliferative advantage. Collectively, dysregulation of midasin has significant consequences for cellular homeostasis and has been implicated in pathological states characterized by aberrant growth, including cancer. Given the central role of translational control in tumor progression and therapy response, alterations in midasin activity may also influence molecular pathways that contribute to resistance to targeted therapies.

Notably, many patients develop resistance to endocrine therapy without loss of ER expression; instead, resistance commonly arises through acquisition of mutations in the estrogen receptor ligand-binding domain (LBD), leading to constitutive receptor activation and diminished responsiveness to antiestrogen therapies (Merenbakh-Lamin et al., 2013; Fanning et al., 2016; Robinson et al., 2013; Toy et al., 2013). These alterations significantly limit the long-term efficacy of many FDA-approved endocrine agents. Although several studies have reported that MDN1 mutations are associated with endocrine resistance (Cornen et al., 2014; Gallegos et al., 2021), high tumor mutational burden and poor prognosis in breast cancer (Hao et al., 2022), they lacked biological validation and did not elucidate a direct mechanistic link between MDN1 alterations and breast cancer progression.

Building on our prior work and emerging evidence implicating midasin, a protein involved in ribosomal biogenesis (Garbarino and Gibbons, 2002; Chen et al., 2018; Prattes et al., 2019), in aromatase inhibitor resistance and recognizing that ESR1 mutations are a defining feature of AI-refractory disease, we sought to determine whether ESR1 mutations cooperate with MDN1 dysregulation to promote tumor adaptation and survival. We therefore hypothesize that mutations in the ER LBD, in conjunction with midasin overexpression, confer properties that enhance and sustain breast cancer cell proliferation. Testing this hypothesis will clarify the functional interplay between mutant ER signaling and ribosome biogenesis machinery and may reveal novel vulnerabilities such as genome instability (Kotina et al., 2025) in TNBC and activating invasion and metastasis through increased stemness (Banjara et al., 2025) in aromatase inhibitor resistant breast cancer.

## 2 Materials and methods

### 2.1 Clinical tumor analysis of MDN1 expression

Midasin protein expression levels were analyzed using The University of Alabama at Birmingham Cancer (UALCAN) data analysis portal (Chandrashekar et al., 2017). The Clinical Proteome Tumor Analysis Consortium (CPTAC) breast cancer protein dataset (accessed on 1 October 2023), and MDN1 was entered into the protein expression query field. Breast cancer subtypes were selected to evaluate differential protein abundance across subtypes. UALCAN generated quantitative box and whisker plots comparing MDN1 protein levels between normal breast tissues and the major molecular subtypes of breast cancer. Statistical significance for differences in protein expression was obtained directly from the UALCAN platform using its Integrated analytical pipeline.

### 2.2 Bioinformatic analysis of MDN1 and ESR1 genomic alterations

An *in silico* analysis was performed using the cBioPortal for Cancer Genomics to explore genomic alterations and their co-occurrence between MDN1 and ESR1 in metastatic breast cancer. The Metastatic Breast Cancer Project (Provisional, December 2021) dataset was queried using the “*Query by Gene”* function, generating an OncoPrint visualizing deep deletions, amplifications, and missense across patients (Cancer Genome Atlas Network, 2012; Cerami et al., 2012; Ciriello et al., 2015; Gao et al., 2013; López-Cortés et al., 2020). A mutual exclusivity and co-occurrence analysis was then conducted using the cBioPortal integrated statistical module, which applies Fisher’s exact test to compute the log odds ratio (LOR) and *p value*.

### 2.3 Cell culture

The MCF-7Luc (wild type; hereafter MCF-7), MCF-7 Y537S (tyrosine is substituted with serine at amino acid position 537), and MCF-7 D538G (substitution of aspartic acid with glycine at position 538) cell lines have been described (Harrod et al., 2022). Briefly, these cells were developed by genome editing that was performed using adeno-associated virus (AAV) technologies to knock-in *ESR1* mutations into MCF7 cell lines as previously described (Harrod et al., 2022). The MCF-7, MCF-7 Y537S, and MCF-7 D538G cells were cultured and maintained in phenol red containing Dulbecco’s Modified Eagle Medium (DMEM), supplemented with 10% fetal bovine serum (VWR, Radnor, PA, United States), 1% antimycotic-antibiotic solution (Thermo Fisher Scientific, Gibco, Waltham, MA, United States), 1% MEM Non Essential Amino Acids (Thermo Fisher Scientific, Gibco, Waltham, MA, United States), 1% MEM Essential Amino Acids (Thermo Fisher Scientific, Gibco, Waltham, MA, United States) 1% sodium pyruvate (Thermo Fisher Scientific, Gibco, Waltham, MA, United States), and 6 μL insulin, human recombinant zinc (Thermo Fisher Scientific, Gibco, Waltham, MA, United States). The MCF-10A cells were cultured and maintained in phenol red containing DMEM/F12 (Thermo Fisher Scientific, Gibco, Waltham, MA, United States) and supplemented with 5% horse serum Thermo Fisher Scientific, Gibco, Waltham, MA, United States), 1% penicillin-streptomycin (Thermo Fisher Scientific, Gibco, Waltham, MA, United States), 50 μL Hydrocortisone (Stem Cell Technologies, Cambridge, MA, United States), 100 μL EGF (Thermo Fisher Scientific, Gibco, Waltham, MA, United States), and 1,250 μL insulin, human recombinant zinc (Thermo Fisher Scientific, Gibco, Waltham, MA, United States). All cells were incubated in a tissue culture environment under a humidified atmosphere of 5% CO_2_ and 95% air at a temperature of 37 °C.

### 2.4 Cell viability studies

Proliferation assays were conducted by seeding cells in a 96-well plate at a density of 7,000 cells per well and allowing them to recover for 24 h. Following the recovery period, the cells were treated with DMSO (vehicle control), Rbin-1 or Rbin-2 (0.1 μM, 1 μM, 3 μM, 6 μM, 12 μM,18 μM) or 33.3 μM 5-Flourouracil (5-FU) for 24, 48, or 72 h. Afterwards, the media was replaced with an MTT solution (0.5 mg/mL) (Sigma-Aldrich, catalog No: M5655-1G), and the absorbance of the resulting solution was measured at 570 nm using a microplate reader (BioTek Synergy H1). The results were analyzed using two-way ANOVA with GraphPad Prism version 10. All experiments were performed with n ≥ 3, and three biological replicates were conducted.

### 2.5 Western blot analysis

The cells were cultured under standard growth conditions until approximately 80%–85% confluency and lysed for protein extraction. For treatment studies, cells were treated with DMSO or Rbin-2 (12 µM) for 48 h, then harvested and centrifuged at 3,500 rpm for 10 min. The resulting cell pellets were resuspended in ice-cold RIPA buffer (catalog # 89900) (Thermo Fisher Scientific, Waltham, MA, United States) supplemented with Halt™ Protease Inhibitor Cocktail, EDTA-Free (100X) (catalog # 78425) (Thermo Fisher Scientific, Waltham, MA, United States). This suspension was then incubated at −80 °C for 3 h to ensure complete lysis, followed by microcentrifugation at 12,000 rpm for 20 min. The bicinchoninic acid (BCA) assay was used to measure the protein concentration. The Laemmli protein sample buffer (Bio-Rad, Hercules, CA, United States) was added to the lysates and heated at 95 °C for 5 min. Protein samples (75–100 μg) were separated using either a 4%–20% Mini-PROTEAN® TGX™ Precast Protein Gel for proteins with a molecular weight <200 kDa or a 7.5% Mini-PROTEAN® TGX™ Precast Protein Gel (Bio-Rad) for proteins >200 kDa. For high molecular weight proteins, gel electrophoresis was conducted for 6 h and 30 min for maximum band separation. Afterwards, the proteins were subsequently transferred to polyvinylidene difluoride (PVDF) membranes and blocked using 5% bovine serum albumin (BSA) in phosphate-buffered saline with 0.1% Tween-20 (PBS-T) for 2 h. The membranes were incubated with primary antibodies specifically targeting ERα (Cell Signaling Technologies, catalog number: 13258S), GAPDH (Cell Signaling Technologies, catalog # 2118L), Vinculin (Cell Signaling Technologies, catalog # 13901S) or MDN1 (Sigma Aldrich, catalog number: HPA029666). Following primary antibody incubation, the appropriate horseradish peroxidase-conjugated secondary antibodies were incubated for 2 h at room temperature. The membranes were washed three times with PBS-T, and protein bands were visualized using Clarity™ western ECL Substrate (Bio-Rad, catalog # 1705061) according to the manufacturer’s instructions. Visualization was achieved with the Chemi Doc XRS system (Bio-Rad), which automatically adjusted the exposure time. Band intensities were quantitatively analyzed using Image Lab software version 6.1 (Bio-Rad). Densitometric analysis was expressed as mean ± standard deviation. Normalization was performed against the housekeeping protein GAPDH or vinculin, adjusting the density of the target proteins compared to the control. The immunoblot images are representative of at least three independent experiments where each was performed with a minimum of two duplicates per sample.

### 2.6 Molecular docking

#### 2.6.1 Ligand preparation

The 3D structures of Rbin-1 and Rbin-2 were retrieved from the PubChem database and prepared using the LigPrep module of the Schrödinger Suite 2024-4. Structures of Rbin-3, Rbin-4, Rbin-5, Rbin-6, and Rbin-7 were generated using ChemDraw and exported as Structure Data Files. The resulting files were used as input for downstream molecular modeling and simulation studies performed with the Schrödinger software suite. Ligand pre-processing included the correction of bond orders, adjustment of bond angles, and confirmation of chiral centers. Energy minimization was performed using the OPLS4 force field to generate low-energy conformers. The ionization and tautomeric states of each ligand were predicted with the Epik module at a physiological pH of 7.0 ± 2.0 to ensure accurate protonation. All remaining parameters were applied as implemented in the Schrödinger Suite to ensure reproducibility of the docking protocol (Patil et al., 2025).

#### 2.6.2 Protein preparation

The crystal structure of midasin was obtained from the UniProtKB database and processed using the Protein Preparation Wizard in Schrödinger Suite 2024-4. Protein pre-processing involved the addition of hydrogen atoms, assignment of bond orders, and removal of water molecules located more than 5 Å from the hetero groups. Disulfide linkages were generated where applicable, and missing loops and side chains were reconstructed using the Prime module. The structure was subsequently optimized and energy-minimized with PROPKA-assigned protonation states at pH 7.4, applying the OPLS4 force field to refine geometry and relieve steric clashes prior to docking.

#### 2.6.3 Grid generation and molecular docking

The binding site grid for midasin was generated by defining the centroid of the predicted ligand-binding pocket as the grid center. Molecular docking of the Rbin compounds was performed using the GLIDE module in extra-precision (XP) mode to maximize scoring accuracy and pose discrimination. The Van der Waals scaling factor and partial charge cut-off were set to 0.80 and 0.15, respectively, to allow for limited flexibility within the active site. Docking calculations were conducted under standard convergence criteria, where the top-ranked poses were evaluated using Glide Score, hydrogen bonding patterns, and hydrophobic interactions to support post-docking visualization and binding analysis.

### 2.7 Statistical analysis

Results are expressed as the mean unit ± standard error of the mean (SEM) (***p < 0.001, **p < 0.01, *p < 0.05) using the Graph Pad Prism V.10 software program. Statistical significance between vehicle control (DMSO) and treatment groups were determined using an unpaired two-tailed Student’s t-test. Comparisons among three groups were analyzed using one-way ANOVA followed by Tukey’s multiple comparisons test. For MTT assays, statistical comparisons were performed using two-way ANOVA followed by Bonferroni’s multiple comparisons test.

## 3 Results

### 3.1 Midasin expression is increased in aggressive breast cancer

**FIGURE 1:**
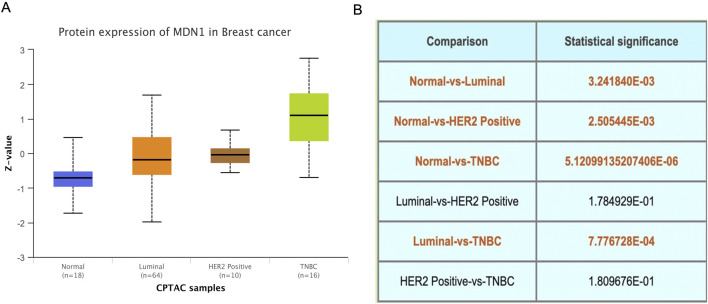
MDN1 protein expression across breast cancer subtypes. **(A)** Boxplot represents the normalized MDN1 expression (Z-scores) in normal (blue), luminal (orange), HER2 positive (brown), and triple-negative (green) patient samples from the CPTAC dataset (left). **(B)** The chart on the right shows the comparison of various samples and their statistical significance (right).

Previous studies from our lab demonstrated that aromatase inhibitor resistant cells cultured three dimensionally (3D) as mammospheres exhibited significantly higher MDN1 expression compared to cells cultured adherently (i.e., two dimensionally), suggesting a potential role for MDN1 in endocrine resistance and cancer stemness (Gallegos et al., 2021). Considering this finding, we sought to determine whether midasin expression was altered across various breast cancer subtypes. To explore this relationship, we utilized the publicly available CPTAC dataset to quantify MDN1 protein expression levels in patients across distinct breast cancer subtypes, including normal, luminal, HER2+, and triple negative breast cancer (TNBC) (Figure 1A). Our analysis revealed that MDN1 expression is higher in all subtypes of breast cancer compared to normal breast tissue. While the statistical comparisons confirmed significant differences between normal tissue and all tumor subtypes except HER2+ (Figure 1B), the dataset consisted of only 10 HER2+ samples which may somewhat skew the interpretation of the clinical significance. The most pronounced increase was observed in TNBC. These findings suggest that MDN1 overexpression may be linked to more aggressive breast tumor phenotypes with acquired or *de novo* endocrine resistance.

### 3.2 Co-occurrence of midasin and ESR1 mutations in breast cancer

**FIGURE 2:**
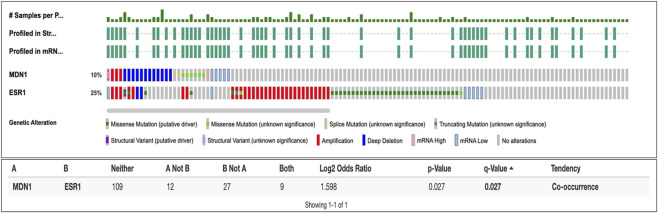
Co-occurrence of MDN1 and ESR1 alterations in breast cancer. MDN1 alterations (10%) and ESR1 alterations (25%) were identified across breast cancer samples. The genetic alterations were missense mutations (putative driver) (dark green squares), missense mutations (unknown significance (light green), amplifications (red rectangles), or deep deletions (blue rectangles).

Given that point mutations in the ER LBD are well-established mechanisms of endocrine resistance, we were interested in identifying whether there was a correlation between ER and midasin mutations. To test this, we analyzed breast cancer patient samples using data from Metastatic Breast Cancer Project (Provisional 2021) dataset in cBioPortal (Figure 2). Our analysis revealed that MDN1 is altered in 10% of the patient samples, primarily through deep deletions and missense mutations, while 25% of the patient’s samples contain amplifications and missense mutations in ESR1. Notably 2.4% of patients exhibit a significant co-occurrence in the genomic alterations of MDN1 and ESR1 (log_2_ odds ratio = 1.598; q = 0.027) which indicates that these alterations frequently appear together in the same tumors more often than expected. The potential genetic interdependence and cooperative nature of the co-occurrence provided a rationale to further explore the significance of this relationship in the *ESR1* mutant clinical setting.

### 3.3 Midasin and ER expression profile in MCF-7 and MCF-7 ER mutant cell lines

**FIGURE 3:**
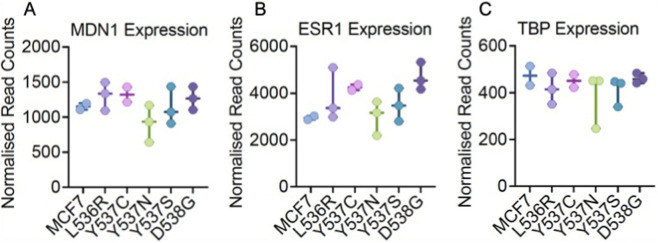
MDN1 is expressed in Y537S and D538G ER mutant MCF-7 Cells. RNA-seq normalized read counts for **(A)** MDN1, **(B)** ESR1, and **(C)** TBP (housekeeping gene). RNA-seq was performed on MCF-7 wild type and MCF-7 ER mutant cells maintained in estrogen-containing media using 6 biological replicates, except Y537S where n = 5. The filled circles indicate the normalized read counts for each of 2 to 3 independent clones. There was no statistically significant difference in the expression of MDN1 or ESR1 at the transcript level.

To explore the relationship between midasin and ER mutations, ER+ MCF-7 breast cancer cell lines expressing mutations in the ER LBD were used as they are commonly associated with endocrine resistance. Initial validation studies were conducted to measure the expression of midasin and ER to confirm the appropriateness of these *in vitro* model systems. As such RNA-sequencing was previously conducted using ER + MCF-7 breast cancer cell lines containing ER LBD point mutants (i.e., L536R, Y537C, Y537N, Y537S, and D538G) as well as the parental MCF-7 cells from which they were derived (Harrod et al., 2022). The RNA transcript levels of MDN1 and ESR1 were assessed and the TATA-binding protein (TBP) was used as a housekeeping reference gene. At the RNA transcript level all cells expressed MDN1 and compared to the MCF-7 cells, those containing ER point mutations had higher or similar levels of MDN1 (Figure 3). When ESR1 RNA transcript levels were examined, all variants had sufficient ESR1 transcript levels that were above parental MCF-7 cells (Figure 3) with very little changes in the expression of the TBP reference gene. While no statistically significant differences were observed, the results indicated that all cell lines expressed adequate levels of MDN1 and ER suggesting that these cells lines containing ER point mutations were appropriate models to assess the functional consequence of MDN1 expression in *ESR1* mutant breast cancer. For the remaining studies, MCF-7 cell lines with point mutations at amino acid positions Y537S and D538G were used as they represent the most common constitutively active point mutations in the LBD that are known to cause resistance to endocrine therapies in breast cancer (Jeselsohn et al., 2014; Chandarlapaty et al., 2016).

**FIGURE 4:**
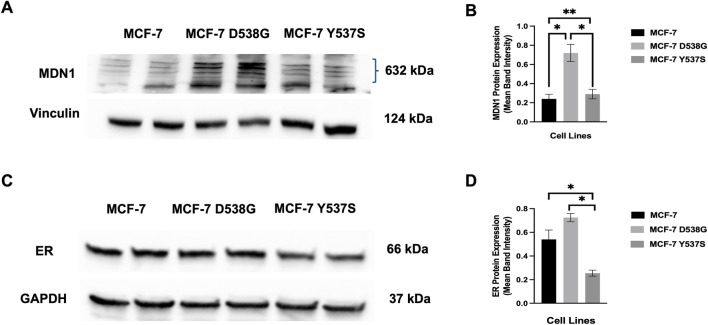
Western blot analysis of MDN1 and ER protein expression in MCF-7, MCF-7 D538G, and MCF-7 Y537S cell lines. **(A)** The immunoblot on the top left depicts the protein expression levels of MDN1 in the MCF-7, MCF-7 D538G, and MCF-7 Y537S cell lines where samples are run in duplicate. Vinculin was measured as an internal loading control. **(B)** Quantitative analysis on the right represents the band intensity of the indicated proteins after normalizing to vinculin loading control. Results are expressed as the mean relative band intensity ± SD (**p < 0.01, *p < 0.05), and data are representative from one of at least three independent experiments. **(C)** The immunoblot on the bottom left depicts the protein expression levels of ER in the MCF-7, MCF-7 D538G, and MCF-7 Y537S cell lines where samples are run in duplicate. GAPDH was measured as an internal loading control. **(D)** Quantitative analysis on the right represents the band intensity of the indicated proteins after normalizing to GAPDH loading control. Results are expressed as the mean relative band intensity ±SD (*p < 0.05), and data are representative from one of at least three independent experiments. All bands were quantified using Image Lab software and band intensities were measured for target protein and corresponding loading controls. The densitometric values are presented as the average ratio of target protein band intensity to loading control band intensity.

To determine whether point mutations in *ESR1* alter MDN1 protein expression, Western blot analyses were conducted in both wild type and *ESR1* mutant breast cancer cell lines. MDN1 protein levels were elevated in *ESR1* mutant cell lines, with the highest expression observed in cells expressing the D538G point mutation, followed by the Y537S mutation, and then the MCF-7 cells (Figures 4A,B). Interestingly, compared to the MCF-7 cells, ERα protein levels were reduced in the cells containing the Y537S mutation and highest in those with the D538G mutation, which may suggest mutation-specific ER stability (Figures 4C,D). Overall, the transcript and protein expression patterns of ER and MDN1 were consistent and preserved across all cell lines.

### 3.4 Computational analyses of a series of ribozinoindole compounds

To evaluate the relevance of these findings *in vitro*, it was critical to first identify a suitable midasin inhibitor that could be used in mammalian cell lines. Based on a previous study Kawashima et al., evaluated a series of seven small molecule MDN1 inhibitors known as ribozinoindoles (Rbin) that were identified as potent, reversible, and selective inhibitors in yeast (Kawashima et al., 2016). Among these, seven analogs were developed and characterized for their inhibitory potential. To determine whether these analogs interacted with the mammalian homolog of midasin, we conducted molecular modeling on the entire series of Rbins analogs. To predict a suitable analog, molecular docking studies were conducted to evaluate the 2D and 3D interaction, interaction type, and docking score (Table 1). The docking simulations resulted in Glide scores that ranged from −6.2 to −2.2 kcal/mol. All analogs exhibited hydrophobic interactions with midasin, while some analogs formed hydrogen bonds, electrostatic interactions, and π-π stacking interactions with midasin.

**TABLE 1:** *In silico* evaluation of the Ribozinoindole series showing ligand-protein interaction patterns and predicted binding affinities.

Compound	3D ligand interaction	2D ligand interaction	Interaction type	Docking score
Rbin-1 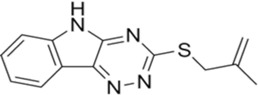	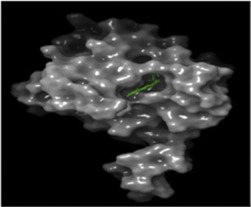	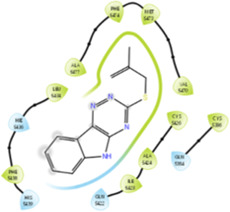	Hydrophobic polar	−6.15 kcal/mol
Rbin-2 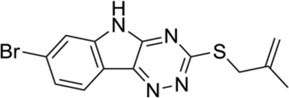	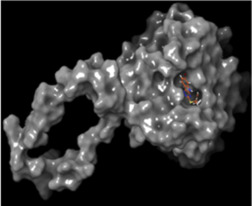	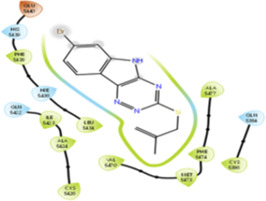	Hydrophobic polar	−5.7 kcal/mol
Rbin-3 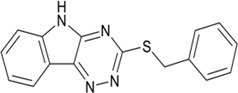	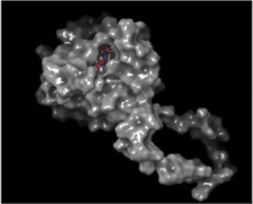	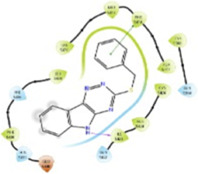	Hydrophobic polar H-Bond Pi-Pi stacking	−5.5 kcal/mol
Rbin-4 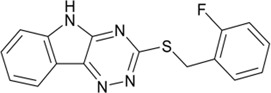	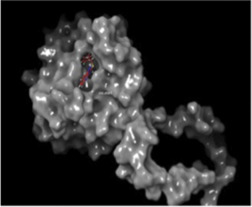	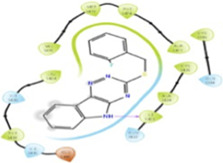	Hydrophobic polar H-Bond	−5.3 kcal/mol
Rbin-5 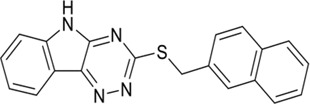	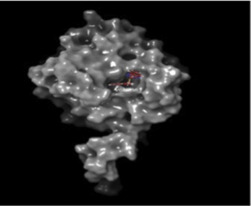	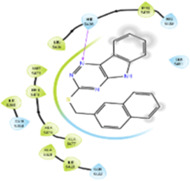	Hydrophobic polar H-Bond	−3.64 kcal/mol
Rbin-6 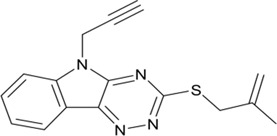	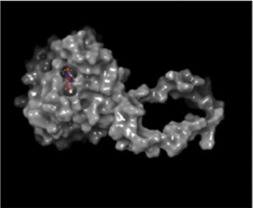	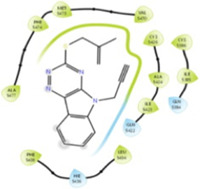	Hydrophobic polar	−4.53 kcal/mol
Rbin-7 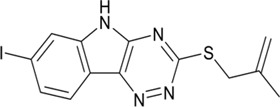	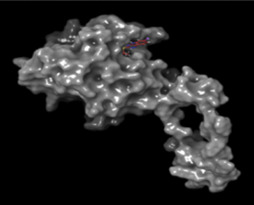	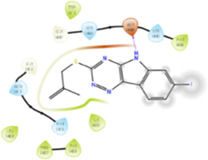	Hydrophobic charged (negative) H-Bond	−2.2 kcal/mol

The table depicts the chemical structures of a series of Rbins analogs, their predicted 3D binding interaction within the target protein, and corresponding 2D interaction maps highlighting residue-level contacts. The types of molecular interactions and predicted docking scores are shown for comparative evaluation of binding affinity across the Rbin series.

The docking simulations indicated that Rbin-2 interacted within a well-defined, surface-accessible pocket on the MDN1 protein. Rbin-2 was primarily stabilized through hydrophobic and polar interactions with key amino acid residues, including Glu 5440, His 5439, Phe 5438, Hie 5436 (i.e., the protonated state of His), Leu 5434, Ala 5477, Phe 5474, Met 5473 and Val 5470, suggesting a favorable binding affinity and orientation within the site. These residues formed a nonpolar microenvironment that accommodates the ligand’s aromatic scaffold, promoting strong hydrophobic stabilization within the cavity. The surrounding polar residues contribute hydrophilic contacts, further supporting ligand orientation and binding affinity. The accompanying 2D interaction diagram illustrated nonpolar contacts and highlighted the overall compatibility between Rbin-2 with the MDN1 binding environment.

The 2D ligand-protein interaction diagram shows that Rbin-1 adopted a well-defined binding pose within the MDN1 binding cavity, stabilized through hydrophobic and hydrophilic interactions. The hydrophobic region, encompassing residues Ser 5469-Ala 5476, forms an extensive nonpolar groove that engages the aromatic core of Rbin-1, promoting compact binding and structural stabilization. In contrast, residues Ile 5423-Ala 5477 contribute hydrophilic contacts, engaging polar atoms on the ligand to reinforce its orientation within the pocket. Overall, the docking simulation yielded a Glide Score of −6.15 and −5.70 for Rbin-1 and Rbin-2 respectively, indicating a similar predicted binding affinity. While Rbin-1 exhibited a slightly greater number of hydrophobic contacts and tighter surface complementarity, both ligands interact within the same general binding pocket of MDN1. Overall, these findings suggest that Rbin-1 adopts a slightly more energetically favorable binding configuration.

### 3.5 Biological evaluation of Rbin-1 and Rbin-2

**FIGURE 5:**
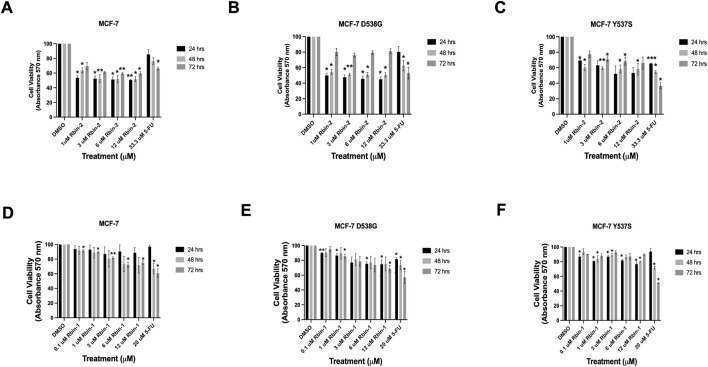
Rbin-1 and Rbin-2 Differentially Effect the Growth of *ESR1* Mutant and Wild Type Cells. **(A)** MCF-7, **(B)** MCF-7 D538G, and **(C)** MCF-7 Y537S cell lines were cultured in standard growth media and treated with DMSO vehicle, 0.1 µM, 1 μM, 3 μM, 6 μM, 12 µM Rbin-2, or 33.3 µM 5 FU or **(D)** MCF-7, **(E)** MCF-7 D538G, and **(F)** MCF-7 Y537S cell lines were cultured in standard growth media and treated with DMSO vehicle, 0.1 µM, 1 μM, 3 μM, 6 μM, and 12 µM Rbin-1 or 33.3 µM 5 FU. All cells were treated for 24, 48, and 72 h and the absorbance was measured at 570 nm as an indicator of cell viability. Results are expressed as the mean relative cell viability ±SD (*p <* 0.05, **p < 0.01, ***p < 0.001), and data are representative from one of at least three independent experiments. Statistical comparisons were performed using two-way ANOVA followed by Bonferroni’s multiple comparisons test.

**FIGURE 6:**
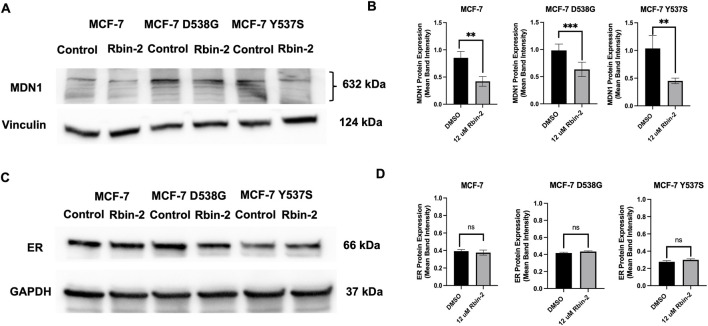
Rbin-2 treatment attenuates midasin expression in MCF-7 parental and ER mutant breast cancer cell lines. The immunoblot on the left depicts the protein expression levels of **(A)** MDN1 or **(C)** ER in the MCF-7, MCF-7 D538G, and MCF-7 Y537S cell lines ±12 μM Rbin-2 treatment for 48 h. Vinculin or GAPDH was measured as an internal loading control. Quantitative analysis on the right represents the band intensity of **(B)** MDN1 and **(D)** ER after normalizing to vinculin loading control. Results are expressed as the mean relative band intensity ±SD (*p < 0.05), and data are representative from one of at least three independent experiments. All bands were quantified using Image Lab software and band intensities were measured for target protein and corresponding loading controls. The densitometric values are presented as the average ratio of target protein band intensity to loading control band intensity. Statistical significance between DMSO and Rbin-2 treatment groups was determined using an unpaired two-tailed Student’s t-test.

While all the analogs successfully docked to the midasin protein, only Rbin-1 and Rbin-2 are commercially available, so they were selected for biological evaluation in our mammalian model systems. To explore the impact of MDN1 on cell proliferation, MTT assays were conducted where the MCF-7, MCF-7 Y537S, and MCF-7 D538G breast cancer cell lines were treated with the midasin inhibitors (Rbin-1 and Rbin-2) at 0.1–12 µM. Cell viability was assessed at 24, 48, and 72 h (Figure 5). In the presence of Rbin-1 there was only a modest reduction in growth. However, when the MCF-7 cells were treated with Rbin-2, there was a (30%–50%) reduction in cell viability, particularly at 24 and 48 h. Similarly, the MCF-7 Y537S cells showed a 35%–45% reduction in viability (Figure 5). In contrast, the MCF-7 D538G cells were more sensitive to lower concentrations of Rbin-2 treatment, with a reduction in viability ranging from 30%–55%. Interestingly, the MCF-7 D538G cells exhibited the highest levels of MDN1 protein expression compared to other cell lines and their growth was more susceptible to midasin inhibition. Based on the proliferation results, Rbin-2 was selected for further assessment. Before proceeding with mechanistic studies, the safety profile of Rbin-2 was assessed by conducting proliferation assays in noncancerous MCF-10A breast epithelial cells and measuring growth after 24, 48 and 72 h (Supplementary Figure S1). Given that there was no significant impact on proliferation, Rbin-2 was utilized as a tool to measure how midasin inhibition impacted the cell biology of ER mutant breast cancer cells. The 12 µM dose was selected as it consistently induced 40%–50% growth inhibition across the cell lines while not causing toxicity. Consequently, Western blotting analyses were conducted to measure the impact and specificity of 12 µM Rbin-2 treatment on midasin protein expression. Results indicated that in all three cell lines, midasin protein expression was reduced by approximately 50% (Figures 6A,B), which suggested that Rbin-2 selectively inhibited midasin protein expression. Next, we measured the effect of Rbin-2 treatment on ER protein expression and there was no significant change in ER expression (Figures 6C,D).

## 4 Discussion

Mutations in the ER ligand-binding domain (LBD) are frequently associated with a constitutively active, hormone-independent phenotype that contributes to endocrine resistance. Cell lines harboring these mutations provide robust models to examine how sustained ER signaling promotes tumor growth in this subtype of breast cancer. Based on our dual observations of co-occurring midasin and ER alterations, together with increased midasin expression in aggressive breast tumor phenotypes, as indicated by the CPTAC results, we hypothesized a functional interplay between midasin and ER signaling. To test this hypothesis, we conducted a series of studies aimed at elucidating the mechanistic basis of this relationship.

Initial experiments focused on identifying an appropriate model system to investigate midasin-ER interactions, followed by evaluating whether Rbins previously identified as midasin inhibitors in yeast exhibit activity in mammalian breast cancer cell lines. Midasin transcript levels were comparable across MCF-7 wild-type cells and MCF-7 cells harboring ER mutations. Given that Y537S and D538G are among the most common constitutively active ESR1 point mutations associated with endocrine resistance (Fribbens et al., 2016), these models were selected for further investigation. Notably, analysis of midasin protein expression revealed mutation-specific differences: midasin was most highly expressed in MCF-7 D538G cells, followed by MCF-7 Y537S cells, and lowest in wild-type cells. These findings suggest a potential mutation-dependent modulation of its expression.

Although wild-type ER protein levels varied among the cell lines, mutant ER is typically dominant and can override wild-type ER signaling (Bahreini et al., 2017), thereby promoting estrogen-independent transcriptional programs and altered downstream signaling. Collectively, these observations underscore that breast cancer cell behavior and therapeutic responsiveness are determined not merely by the presence of wild-type ER, but by the relative abundance and functional interplay between wild-type and mutant ER.

To this end, we hypothesized that mutations in the ER LBD, in combination with midasin overexpression, confer properties that enhance and sustain breast cancer cell proliferation. Accordingly, we anticipated that cells harboring ER LBD point mutations would exhibit greater sensitivity to the anti-proliferative effects of midasin inhibition. However, both MCF-7 wild-type and MCF-7 Y537S mutant cells demonstrated similar responses to Rbin-2 treatment. This comparable proliferative response may be explained by two factors. First, the findings suggest that, irrespective of ER mutational status, cells remain fundamentally dependent on basal MDN1 activity for survival and proliferation which accounts for the Rbin-2 mediated growth inhibition in all cells. The global dependency on midasin for growth is also supported by our proliferation studies in various breast cancer settings including ER^low/-^ (Banjara et al., 2025; Gallegos et al., 2021) and TNBC (Kotina et al., 2025). Second, differences in the relative expression of wild-type and mutant ER transcript likely contribute to this observation. Specifically, MCF-7 D538G cells predominantly express the mutant receptor transcript, whereas MCF-7 Y537S cells retain a higher proportion of wild-type ER. Consequently, MCF-7 Y537S cells exhibit phenotypes more similar to the wild-type cells due to their higher wild-type receptor ratio. Taken together, these results suggest a cooperative interaction between ESR1 mutations and midasin expression in regulating breast cancer cell proliferation. Future studies are underway to examine dual targeting of midasin and ER mutants.

After validating the suitability of the selected cell lines for this study, we conducted computational docking analyses using a panel of Rbin analogs. These analyses yielded comparable docking scores and highly similar predicted interaction profiles across all seven compounds. Notably, the Rbin analogs were consistently predicted to interact to the C-terminal region of midasin, corresponding to the location of the Metal Ion Dependent Adhesion Site (MIDAS) domain. This observation contrasts with a previous model proposing that Rbins inhibit midasin through interactions with its ATPases Associated with diverse cellular Activities (AAA+) ATPase region, thereby impairing ATP-dependent remodeling of pre-60S ribosomal particles (Kawashima et al., 2016). Although this alternative binding mode is mechanistically plausible, a key limitation of this study is that both docking, and interaction analyses are based on *in silico* simulations and therefore serve as predictive rather than definitive evidence of binding. In addition, the absence of a well-characterized reference compound for benchmarking the Rbin analogs introduces uncertainty and allows for alternative interpretations of these results. Despite these limitations, a previous study challenged the concept the Rbin-1 targeted the AAA-ATPase domains exclusively but rather acts as an allosteric inhibitor (Mickolajczyk et al., 2020). Instead, it was proposed that Rbin-1 tethers MIDAS to the AAA-ATPase domain to relieve the regulatory effects of the linking domains (Mickolajczyk et al., 2020). Further support of this model and our findings is the consistent localization of all analogs to the C-terminal region of midasin reduces the likelihood that this finding is attributable to random binding or modeling artifacts, supporting the potential biological relevance of this interaction.

Although the precise binding site of Rbins has not yet been structurally defined, our data raise the possibility of a previously unrecognized interaction site within the MIDAS domain. This hypothesis is mechanistically feasible, as the MIDAS domain mediates the metal-dependent extraction of assembly factors from pre-60S ribosomal particles, a process essential for late-stage 60S ribosomal subunit maturation. Disruption of this domain would be expected to result in the accumulation of immature pre-60S particles, impaired ribosomal subunit export, reduced global protein synthesis, and activation of cellular stress responses. Future studies will be required to experimentally define the precise binding site and mode of action of Rbin.

**FIGURE 7:**
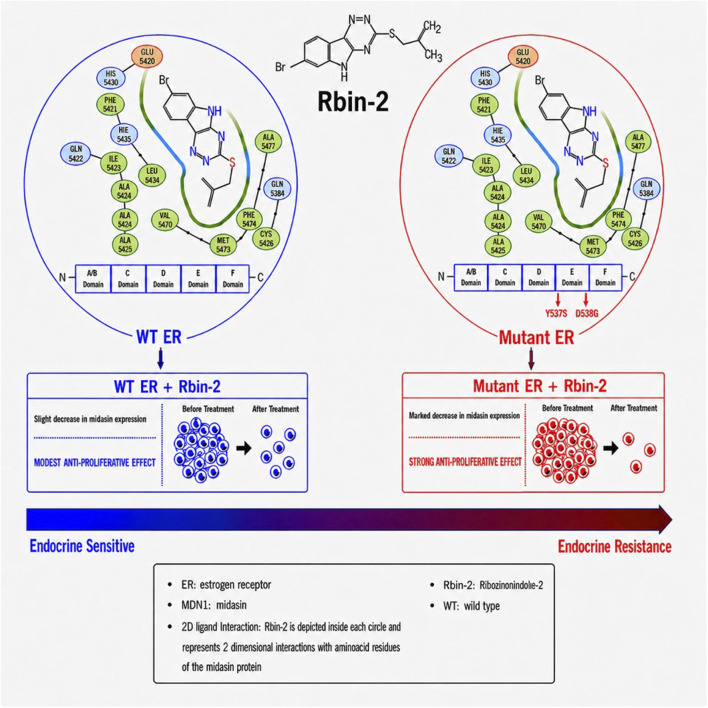
Proposed model illustrating the net effect of Rbin-2-mediated midasin inhibition on endocrine-resistant breast cancer cells. Endocrine-sensitive MCF-7 breast cancer cells (red) express wild-type estrogen receptor (ER) and exhibit low levels of midasin. In contrast, endocrine-resistant MCF-7 D538G cells (blue) harbor activating mutations in the ER and display elevated midasin expression. The MCF-7 Y537S cells may represent a transitory state between the MCF-7 and MCF-7 D538G cells. As breast cancer cells acquire additional ER mutations and progress toward more advanced endocrine resistance, dependence on midasin increases. Inhibition of midasin by Rbin-2 disrupts ribosome biogenesis through binding to the C-terminal domain of the midasin protein, resulting in reduced cellular proliferation. Consequently, cells with high midasin expression and mutant ER are more sensitive to the antiproliferative effects of Rbin-2, whereas endocrine-sensitive cells with lower midasin expression and wild-type ER exhibit reduced sensitivity to midasin inhibition.

In conclusion, functional studies assessing the effects of Rbin analogs on cell viability and protein expression demonstrate that, although Rbin-1 displayed a more favorable predicted binding configuration, Rbin-2 exhibited greater biological efficacy. These findings indicate that midasin inhibition differentially affects cellular proliferation in an ER mutation-dependent manner. Specifically, cells harboring the Y537S mutation, which express lower levels of midasin and a higher ratio of wild type to mutant ER, were less sensitive to the growth-inhibitory effects of Rbin-2 than cells carrying the D538G mutation which contain higher ratios of the mutant ER and higher midasin expression (Figure 7). Given the heterogeneity inherent to these cells future studies will require investigating multiple clones. However, it is also plausible that midasin is required for growth in this setting and partially co-dependent on ER status and context. Collectively, these results support a positive association between midasin expression and endocrine resistance and suggest that midasin may represent a novel therapeutic vulnerability in treatment-refractory tumors. While the mechanistic relationship between midasin and mutant ER signaling remains to be fully elucidated, as we did not investigate the full impact of MDN1 inhibition on ER signaling, this study establishes the feasibility of pharmacologically targeting midasin in mammalian systems and provides a foundation for future investigations into its role in ER-mutant breast cancer. To this end, studies are currently underway to evaluate how targeting mutated forms of the ER impact endocrine resistance. Despite the limitations, this foundational study establishes the feasibility of pharmacologically targeting midasin in mammalian systems and provides a basis for future investigations into its role in ER-mutant breast cancer.

## Data Availability

The raw data supporting the conclusions of this article will be made available by the authors, without undue reservation.
