# Supplementary material for: Pharmacologic targeting of midasin (MDN1) reveals a potential therapeutic vulnerability in ESR1-mutant breast cancer
Source: Front Pharmacol. 2026 Jul 17;17:1852788. doi: 10.3389/fphar.2026.1852788 (PMC13424217; doi:10.3389/fphar.2026.1852788)
Supplement: Supplementary file 1 [file Presentation1.pptx]

## Slide 1
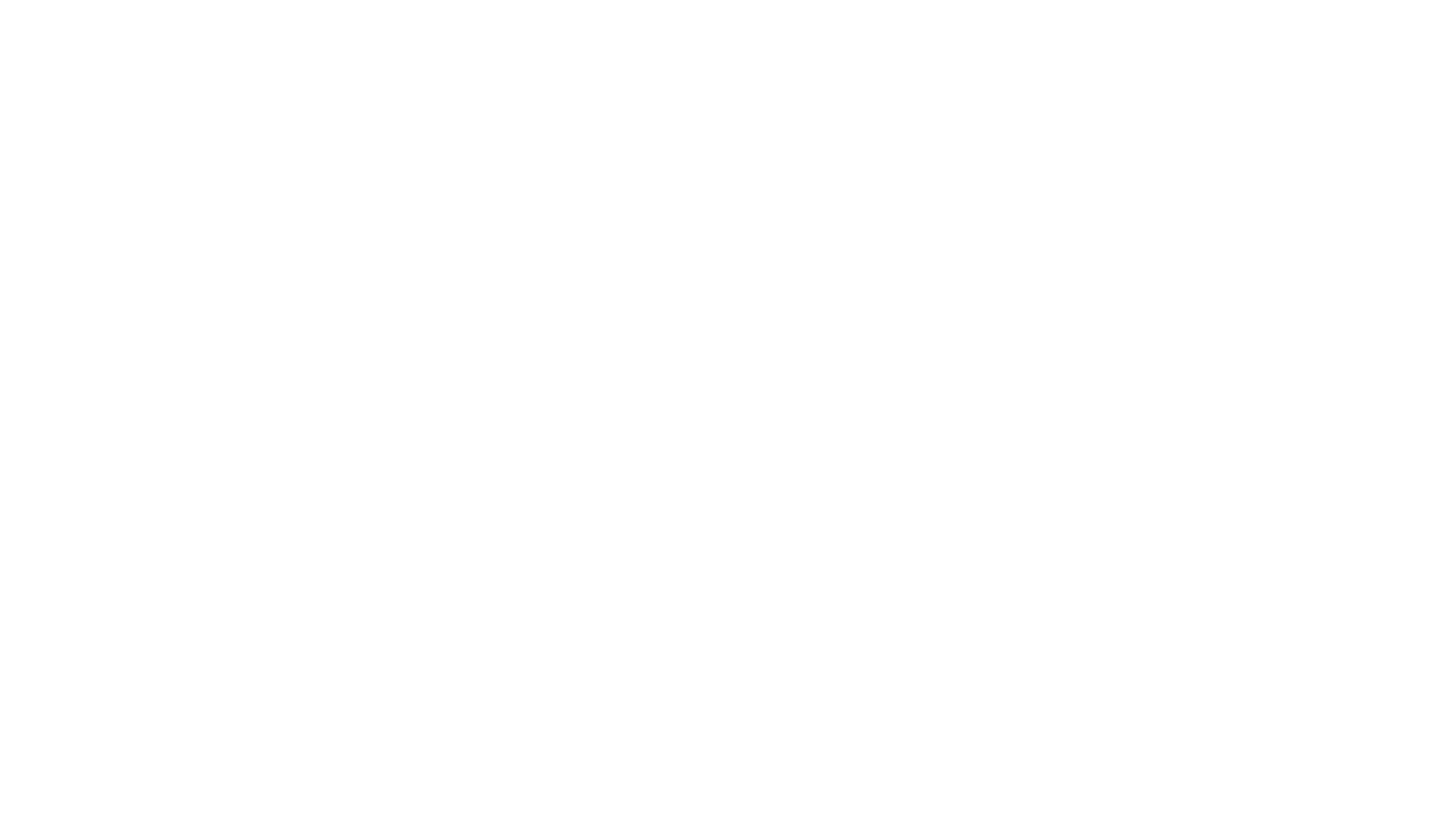

## Slide 2
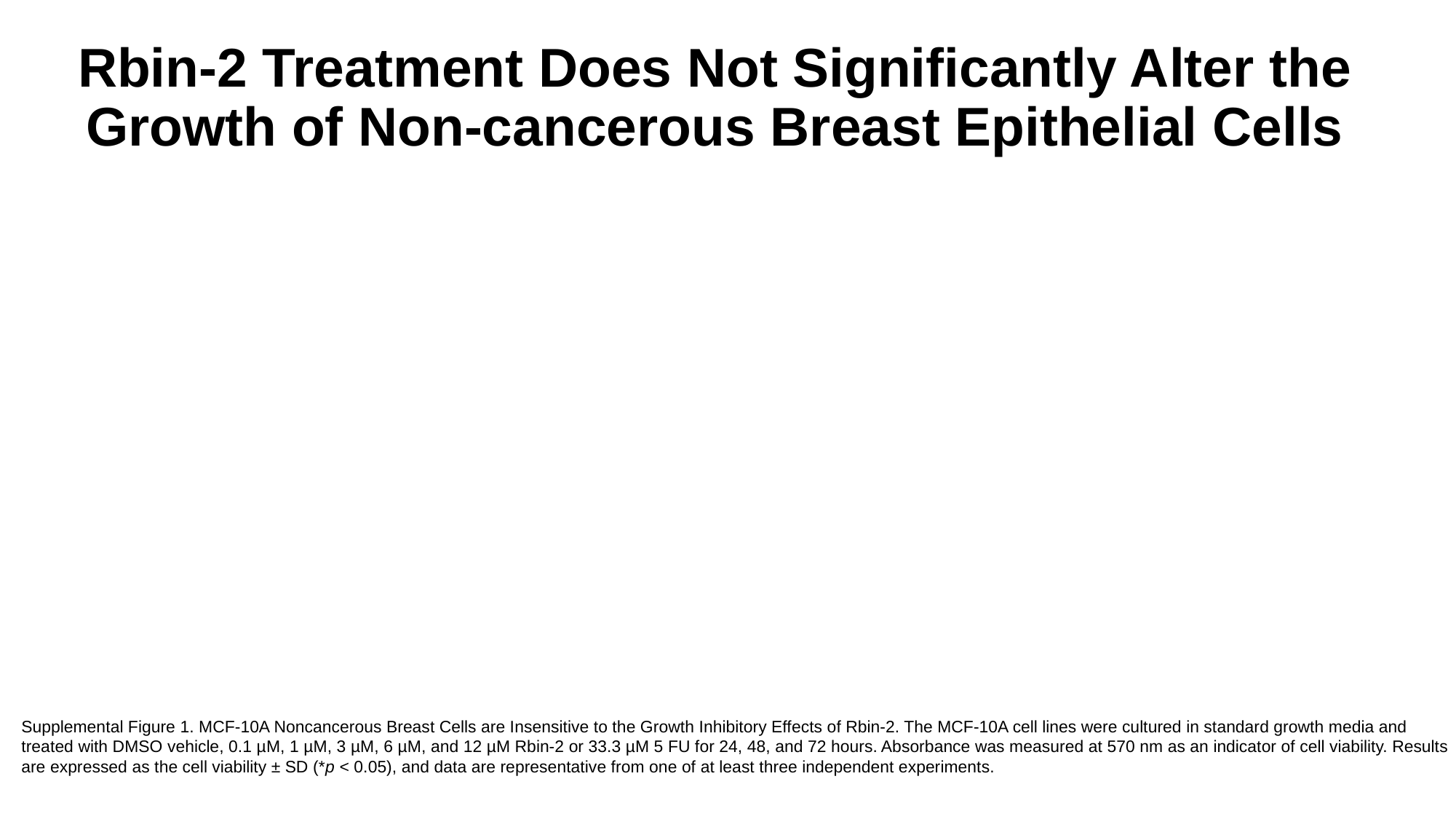

# Rbin-2 Treatment Does Not Significantly Alter the Growth of Non-cancerous Breast Epithelial Cells
Supplemental Figure 1. MCF-10A Noncancerous Breast Cells are Insensitive to the Growth Inhibitory Effects of Rbin-2. The MCF-10A cell lines were cultured in standard growth media and treated with DMSO vehicle, 0.1 µM, 1 µM, 3 µM, 6 µM, and 12 µM Rbin-2 or 33.3 µM 5 FU for 24, 48, and 72 hours. Absorbance was measured at 570 nm as an indicator of cell viability. Results are expressed as the cell viability ± SD (*p < 0.05), and data are representative from one of at least three independent experiments.
